# Statin adherence among patients with hypertension in Swedish primary health care: a QregPV cohort study using explainable machine learning

**DOI:** 10.1093/ehjcvp/pvag048

**Published:** 2026-06-29

**Authors:** Johan Sandslätt, Kristina Bengtsson Boström, Maria Åberg, Johan-Emil Bager, Koen Simons, Georgios Mourtzinis, Per Hjerpe, Tobias Andersson

**Affiliations:** Närhälsan Krokslätt Health Care Centre, Fredåsgatan 13, 431 67 Gothenburg, Sweden; General Practice/Family Medicine, School of Public Health and Community Medicine, Institute of Medicine, Sahlgrenska Academy, University of Gothenburg, 405 30 Gothenburg, Sweden; General Practice/Family Medicine, School of Public Health and Community Medicine, Institute of Medicine, Sahlgrenska Academy, University of Gothenburg, 405 30 Gothenburg, Sweden; Regionhälsan R&D Centre, Skaraborg Primary Care, 541 80 Skövde, Sweden; General Practice/Family Medicine, School of Public Health and Community Medicine, Institute of Medicine, Sahlgrenska Academy, University of Gothenburg, 405 30 Gothenburg, Sweden; Region Västra Götaland, Regionhälsan, 405 30 Gothenburg, Sweden; Department of Emergency Medicine, Sahlgrenska University Hospital, 413 46 Gothenburg, Sweden; Department of Molecular and Clinical Medicine, Institute of Medicine, Sahlgrenska Academy, University of Gothenburg, 413 45 Gothenburg, Sweden; Biostatistics, School of Public Health and Community Medicine, Institute of Medicine, Sahlgrenska Academy, University of Gothenburg, 405 30 Gothenburg, Sweden; Department of Molecular and Clinical Medicine, Institute of Medicine, Sahlgrenska Academy, University of Gothenburg, 413 45 Gothenburg, Sweden; Department of Medicine and Emergency Care Mölndal, Sahlgrenska University Hospital, 431 80 Mölndal, Sweden; General Practice/Family Medicine, School of Public Health and Community Medicine, Institute of Medicine, Sahlgrenska Academy, University of Gothenburg, 405 30 Gothenburg, Sweden; Regionhälsan R&D Centre, Skaraborg Primary Care, 541 80 Skövde, Sweden; General Practice/Family Medicine, School of Public Health and Community Medicine, Institute of Medicine, Sahlgrenska Academy, University of Gothenburg, 405 30 Gothenburg, Sweden; Regionhälsan R&D Centre, Skaraborg Primary Care, 541 80 Skövde, Sweden

**Keywords:** Cardiovascular diseases, Preventive care, Treatment adherence, Statins, Hyperlipidaemia, Primary health care

## Abstract

**Aims:**

Evidence regarding statin adherence among patients with hypertension in primary care is limited. We assessed statin adherence in Swedish primary care and examined associations between patient characteristics and adherence.

**Methods and results:**

In this retrospective register-based cohort study from a large Swedish region, data from all primary health care centres were linked to data on comorbidities, dispensed drugs, and socioeconomic factors. Patients with hypertension who initiated statin therapy in 2012–2015 were included. Adherence during 2 years after initiation was measured using the proportion of days covered (PDC). Explainable machine learning with XGBoost and SHAP values identified baseline characteristics associated with adherence. Associations were estimated using adjusted multilevel regression and presented as odds ratios (ORs) and median odds ratios (MORs). Among 30 497 patients (mean age 66 years; 48% women), the mean 2-year PDC was 73.2%, while 60.2% achieved adequate adherence (PDC > 80%). Adequate adherence differed by indication (*P* < 0.001): 54.0% in primary prevention, 61.3% in diabetes without cardiovascular disease, and 65.5% in secondary prevention. The five most important characteristics associated with adequate adherence according to SHAP values were dispensed antithrombotic therapy (OR 1.38, 95% CI 1.26–1.50), primary health care centre (MOR 1.16, 95% CI 1.12–1.20), 10-year increase in age (OR 1.14, 95% CI 1.12–1.17), initiation with atorvastatin (OR 1.33, 95% CI 1.24–1.44), and countries of birth outside Sweden (OR range 0.73–0.78).

**Conclusion:**

Only 60% of patients achieved adequate statin adherence, which varied substantially by indication. Patient- and provider-level factors identified could guide targeted interventions to optimize preventive cardiovascular care.

## Graphical Abstract

**Graphical Abstract:**
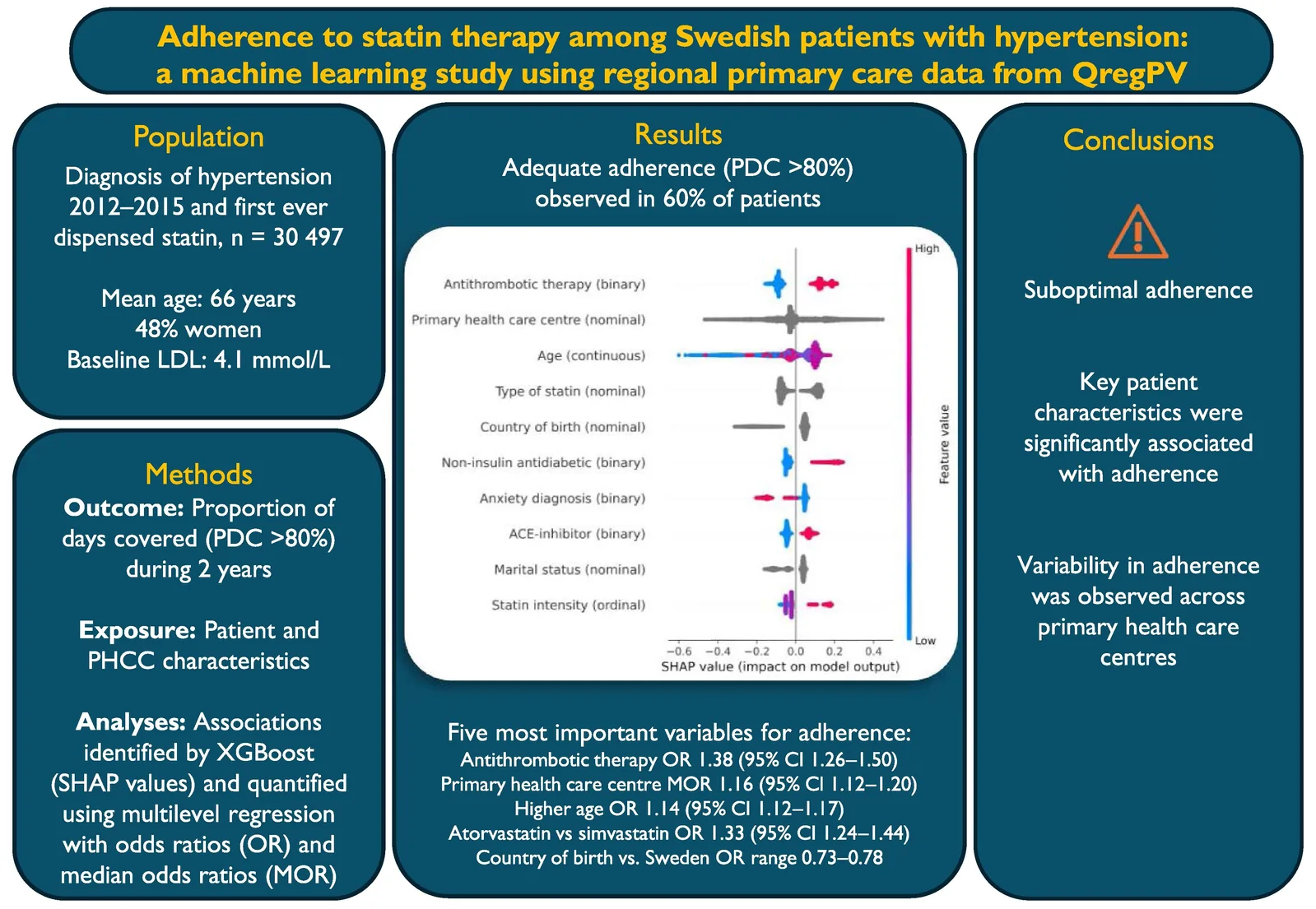

## Introduction

Cardiovascular disease (CVD) remains the leading cause of death in Sweden and globally,^1^ highlighting the need for sustained preventive measures. A meta-analysis of 44 studies involving 2 million patients estimated that ∼ 9% of cardiovascular events in Europe could be attributed to poor adherence to cardiovascular medications.^2^ Despite the proven efficacy,^3,4^ widespread availability, and low cost of statins, adherence remains suboptimal.^5^ This is particularly concerning given that discontinuation or interruption of statin therapy increases cardiovascular risk and mortality.^6,7^

Adherence to statin therapy in secondary prevention is well documented.^8,9^ However, less is known about adherence among patients with hypertension in primary health care, particularly those in primary prevention without concomitant diabetes mellitus (DM) or CVD. To our knowledge, no study has directly compared adherence to statin therapy in hypertensive patients in primary care with and without concomitant DM or CVD. Furthermore, there is limited research on adherence over extended follow-up periods, and explainable machine learning has not previously been applied to investigate how patients’ baseline characteristics are associated with statin adherence among patients with hypertension in a primary health care setting.

Therefore, the aim of this study was to assess 2-year adherence to statin therapy in patients with hypertension in primary care, with and without concomitant DM or CVD, and to identify baseline characteristics associated with adherence using machine learning techniques.

## Methods

### Study design and data sources

This retrospective, register-based cohort study investigated adherence to statin therapy among patients with hypertension in primary health care. The study was conducted in the Swedish Region Västra Götaland (VGR), consisting of ∼ 1.8 million people in 2025, encompassing both urban and rural areas. All primary health care centres (PHCCs) in VGR contribute data to the regional quality-of-care register for primary care, QregPV. This register includes data since 2010 on patients diagnosed with hypertension (ICD-10 codes: I10–I15), DM (E10–E14), and ischaemic heart disease (I20–I25).^10^ In this study, longitudinal data up to 2017 were available on age, sex, smoking status, weight, height, body mass index (BMI), systolic blood pressure, diastolic blood pressure, total cholesterol, low-density lipoprotein cholesterol (LDL-C), and triglycerides. As previously described,^10^ additional regional and national register data on comorbidities, dispensed medications, and socioeconomic variables were linked using Sweden’s unique personal identification number, assigned to all residents (see data sources in Supplementary material).

### Participants

**Figure 1:**
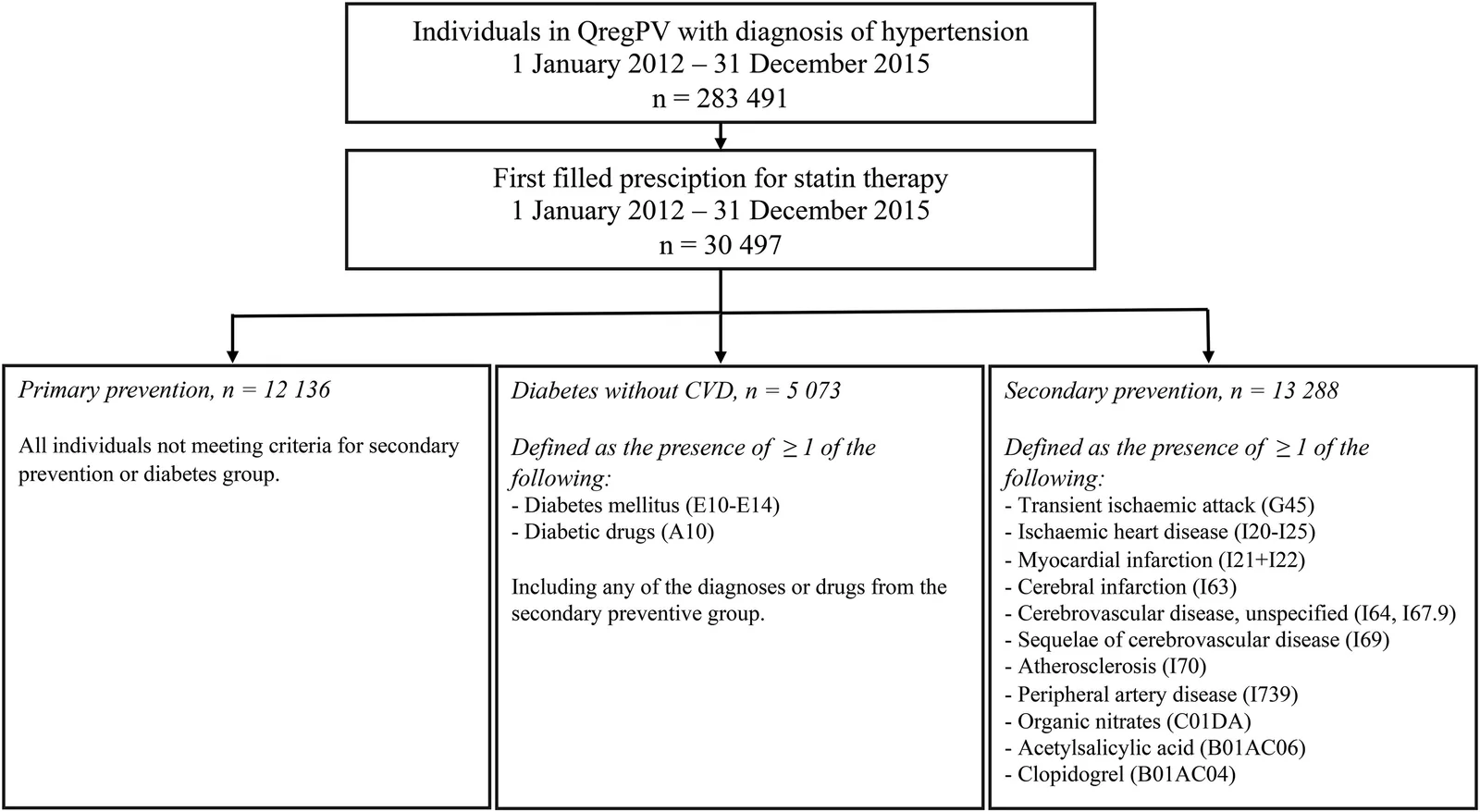
Study flowchart. Among patients classified as receiving secondary preventive therapy, 1657 were classified based solely on secondary prevention diagnoses, 4664 based solely on secondary prevention medications, and 6967 had both a secondary prevention diagnosis and medication.

Patients registered in QregPV with a diagnosis of hypertension (ICD-10 codes: I10–I15), who were dispensed their first statin prescription (ATC code: C10AA) between 1 January 2012 and 31 December 2015, were included in the study. Patients with prior dispensed statin prescriptions (data from 2008) were excluded. Participants were categorized into three subgroups based on comorbidities and dispensed medications: primary prevention, DM without CVD, and secondary prevention. Patients were assigned to the secondary prevention subgroup based on any diagnoses or dispensed medications on or before the date of first statin dispensation, as presented in the study flowchart (*Figure 1*).

### Outcome variables

The study outcome was adherence to statin therapy 2 years after initiation. Adherence was measured using the proportion of days covered (PDC). It takes into consideration gaps in medication availability within the observation period and is calculated as the total days with statin supply divided by the total days in the observation period. PDC was calculated using continuous multiple-interval measures of medication availability (CMA), a commonly used method for quantifying longitudinal medication use.^11^ Among the nine available CMA variants, CMA6 was applied to estimate PDC. CMA6 caps adherence at 100%, carries forward surplus medication for the same statin, and adjusts for changes in dosage.^11^ The observation period began on the date of the first dispensed statin prescription and ended 2 years later or at the time of death, whichever occurred first. Adherence was assessed both as a binary measure, defined as PDC > 80% vs. ≤ 80%, and as PDC as a continuous variable.^12^ In addition, LDL-C was assessed using the most recent values within 1 and 2 years after initiation of therapy, across the three study subgroups and PDC categories. Also, the proportions of patients achieving LDL-C targets in use during the study inclusion period were calculated.^13^ As a descriptive sensitivity analysis, we also calculated adherence > 80% using CMA2, which corresponds to the medication possession ratio (MPR). Unlike the primary adherence measure, CMA2 may yield estimates exceeding 100%.

### Statistical analysis

Differences in unadjusted adherence between the three study subgroups (primary prevention, diabetes without CVD, and secondary prevention), calculated as PDC > 80%, were assessed using Pearson’s χ^2^ test. Differences in unadjusted adherence between the three subgroups were also calculated with PDC as a continuous measure, using the Kruskal–Wallis test. Statin therapy was categorized as low-, moderate-, or high-intensity according to the prescribed dose.^14^ Machine learning with Extreme Gradient Boosting (XGBoost) combined with explainable artificial intelligence using SHapley Additive exPlanations (SHAP) values,^15,16^ were used to identify and visualize the baseline characteristics (see Supplementary material online, *Table S1*) most strongly associated with adherence, defined as PDC > 80%, and in sensitivity analyses as continuous PDC. To estimate the magnitude of the associations between baseline characteristics and adherence, we used multilevel logistic regression models with patients clustered within PHCCs. We estimated unadjusted and adjusted odds ratios (ORs) and, if applicable, median odds ratios (MORs),^17^ for the 10 baseline characteristics most strongly associated with PDC > 80%, as identified by SHAP values. Adjusted ORs were obtained by fitting 10 models with variable-specific adjustment sets determined via the modified disjunctive cause criterion.^18^ For additional details, see Statistical analysis in Supplementary material. All statistical analyses were conducted using R (version 4.4.2) and Python (version 3.12) within RStudio (version 2025.09.1). Adherence measures were calculated using the AdhereR package for R.

### Ethical approval

The study was approved by the Regional Ethical Review Board in Gothenburg (registration numbers: 1062-15, T313-16, T533-18) and the Swedish Ethical Review Authority (registration numbers: 2020-02839 and 2024-06040-02). Informed consent was waived due to the retrospective, register-based design.

## Results

Between 1 January 2012 and 31 December 2015, a total of 283 491 patients were registered with a diagnosis of hypertension in QregPV. Among these, 30 497 patients dispensed their first statin prescription and were included in the study. Of those, 12 136 (40%) were categorized as receiving primary preventive statin therapy, 5073 (17%) had DM without CVD, and 13 288 (43%) were classified as receiving secondary preventive statin therapy.

### Patient characteristics

Baseline characteristics of the study population are presented in *Table 1*, and in Supplementary material online, *Table S1*, including socioeconomic variables, comorbidities and filled prescriptions of cardiometabolic medications within 12 months. The mean age was 66 years, and 48% were women. Patients in the secondary prevention group were older (mean age 71 years) compared to those in the primary prevention and diabetes groups (both with a mean age of 63 years). Mean untreated baseline LDL-C levels were highest in the primary prevention group, 4.5 mmol/L, compared to 3.8 mmol/L in both the diabetes and secondary prevention groups. Details on the type of dispensed statins are presented in Supplementary material online, *Table S2*. Patients in the secondary prevention group were more frequently initiated on high-dose statins compared to those in the primary prevention group. Specifically, simvastatin 40 mg was dispensed to 27.9% of the patients in the secondary prevention group vs. to 12.3% in the primary prevention group, while atorvastatin 80 mg was dispensed to 14.4% vs. 2.8%, respectively.

**Table 1:** Baseline characteristics of individuals included in the study

	Overall	Primary prevention	Diabetes without CVD	Secondary prevention	Missing (%)
Number of individuals	30 497	12 136	5 073	13 288	
Age, years (SD)	66.3 (11.5)	62.5 (10.2)	62.5 (10.5)	71.2 (11.0)	0.0
Women, *n* (%)	14 565 (47.8)	6 330 (52.2)	2 189 (43.2)	6 046 (45.5)	0.0
Weight, kg (SD)	84.4 (18.5)	83.4 (17.6)	92.7 (19.7)	81.7 (17.8)	14.9
Height, cm (SD)	170.5 (9.8)	170.7 (9.6)	171.6 (9.9)	169.9 (9.9)	17.0
BMI, kg/m^2^ (SD)	29.0 (5.3)	28.5 (4.9)	31.4 (5.8)	28.3 (5.2)	19.1
Waist circumference, cm (SD)	101.1 (13.6)	99.1 (13.0)	107.1 (13.7)	100.1 (13.3)	27.2
Total cholesterol, mmol/L (SD)	6.1 (1.3)	6.6 (1.2)	5.6 (1.2)	5.7 (1.2)	16.5
LDL-cholesterol, mmol/L (SD)	4.1 (1.0)	4.5 (0.9)	3.8 (0.8)	3.8 (0.9)	23.3
Triglycerides, mmol/L (SD)	1.8 (1.2)	1.9 (1.2)	2.0 (1.3)	1.7 (1.0)	22.8
SBP, mmHg (SD)	140.3 (17.9)	141.7 (17.5)	136.8 (16.8)	140.4 (18.5)	4.7
DBP, mmHg (SD)	82.8 (12.3)	85.7 (11.9)	81.5 (12.0)	80.6 (12.3)	4.8
HbA1c, mmol/mol (SD)	49.4 (15.1)	38.0 (4.7)	53.0 (15.6)	49.4 (14.9)	68.5^a^
Smoker, *n* (%)	4403 (17.8)	1897 (18.9)	756 (16.0)	1750 (17.5)	18.7

Categorical variables are presented as numbers (%) and continuous variables as means (SD) unless stated otherwise. BMI, body mass index; CVD, cardiovascular disease; SD, standard deviation; SBP, systolic blood pressure; DBP, diastolic blood pressure.

^a^10% missing in the diabetes without CVD group.

### Adherence to statin therapy

The overall mean PDC over the 2-year follow-up period was 73.2% (Supplementary material online, *Table S3*). Patients receiving secondary preventive statin therapy had the highest mean PDC (76.7%), followed by those with diabetes without CVD (74.6%) and lowest among those receiving primary preventive therapy (68.6%) (*P* < 0.001). Likewise, adequate adherence, defined as PDC > 80%, differed by prevention category: 54.0% in the primary prevention group, 61.3% among patients with diabetes without CVD, and 65.5% in the secondary prevention group (*P* < 0.001). Follow-up data of LDL-C is presented in Supplementary material online, *Table S4*. Increasing adherence was associated with a stepwise greater reduction in LDL-C, both in the overall cohort and across all subgroups. The differences between LDL-C at baseline and 2 years after initiation of therapy were all statistically significant. The proportions of patients achieving specific LDL-C targets are presented in Supplementary material online, *Table S5*. In descriptive analyses, the proportion of patients achieving LDL-C targets appeared to be higher with higher levels of adherence across all prevention categories at both 1 and 2 years after initiation of statin therapy. Adherence calculated as a sensitivity analysis, with the MPR-like measure CMA2, yielded higher adherence as expected (see Supplementary material online, *Table S6*).

### Baseline characteristics and adherence

**Figure 2:**
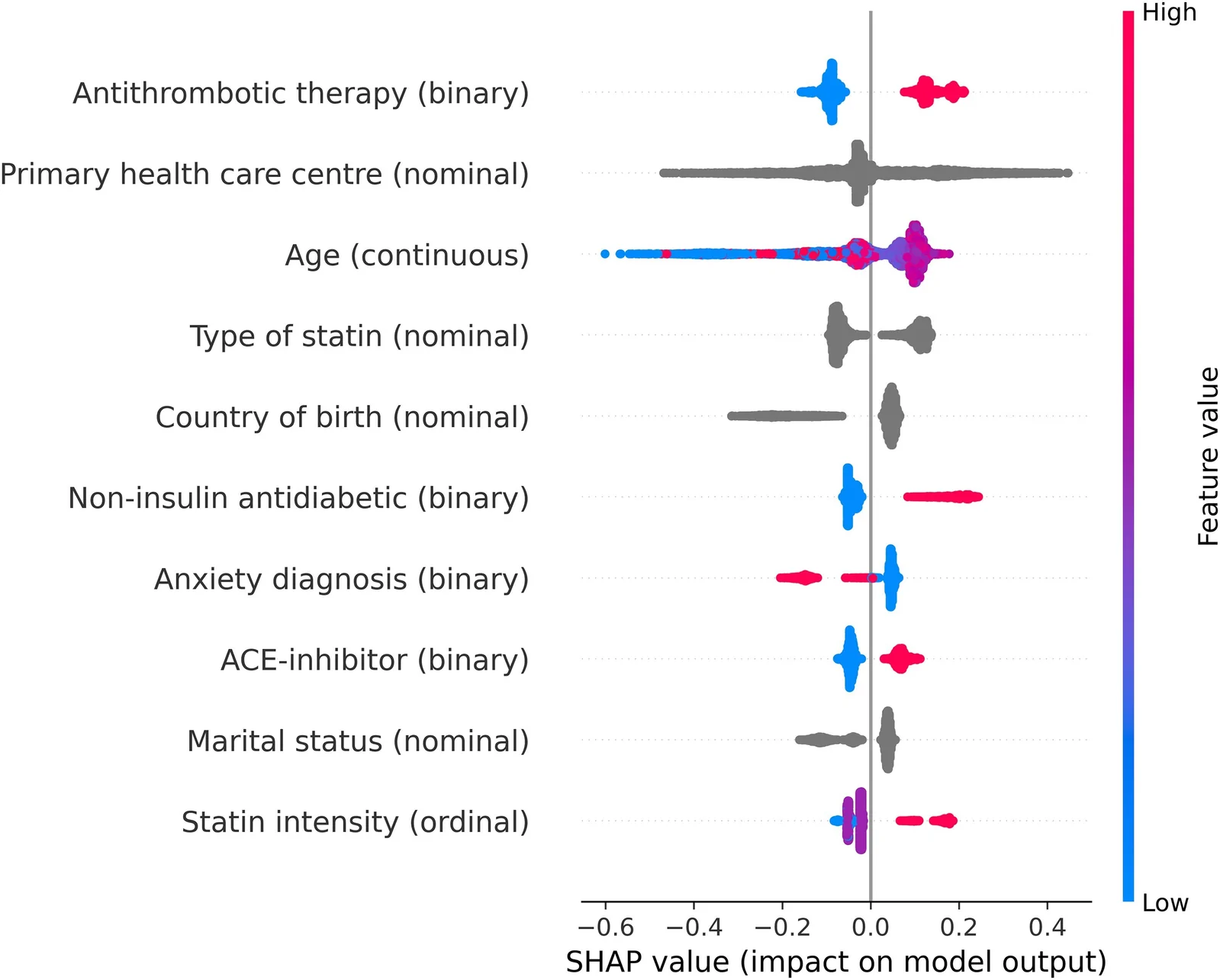
SHAP values for the full study cohort (test data) illustrating the 10 baseline characteristics most strongly associated with predicted adherence, defined as proportion of days covered (PDC) > 80%. Each dot represents one individual. The x-axis shows how much each variable contributed to the prediction of adherence in the machine learning model. Positive SHAP values indicate higher predicted adherence, whereas negative values indicate lower predicted adherence. The colours indicate whether the value of the variable was high (red) or low (blue). ACE: angiotensin-converting enzyme.

Overall, for the whole cohort, the 10 most influential baseline characteristics associated with PDC > 80%, as identified using machine learning with XGBoost and SHAP values, are presented in *Figure 2*. Among those, dispensed antithrombotic therapy, the patients’ PHCCs, age, type of statin, and country of birth were ranked as the five most important baseline variables. The area under the receiver operating characteristic curve (AUC-ROC) score was 0.67 (95% CI 0.66–0.67) in the training set and 0.63 (95% CI 0.62–0.65) in the test set in the full XGBoost model. The final optimized hyperparameter values are presented in Supplementary material online, *Table S7* and calibration plots in Supplementary material online, *Figure S1*.

**Figure 3:**
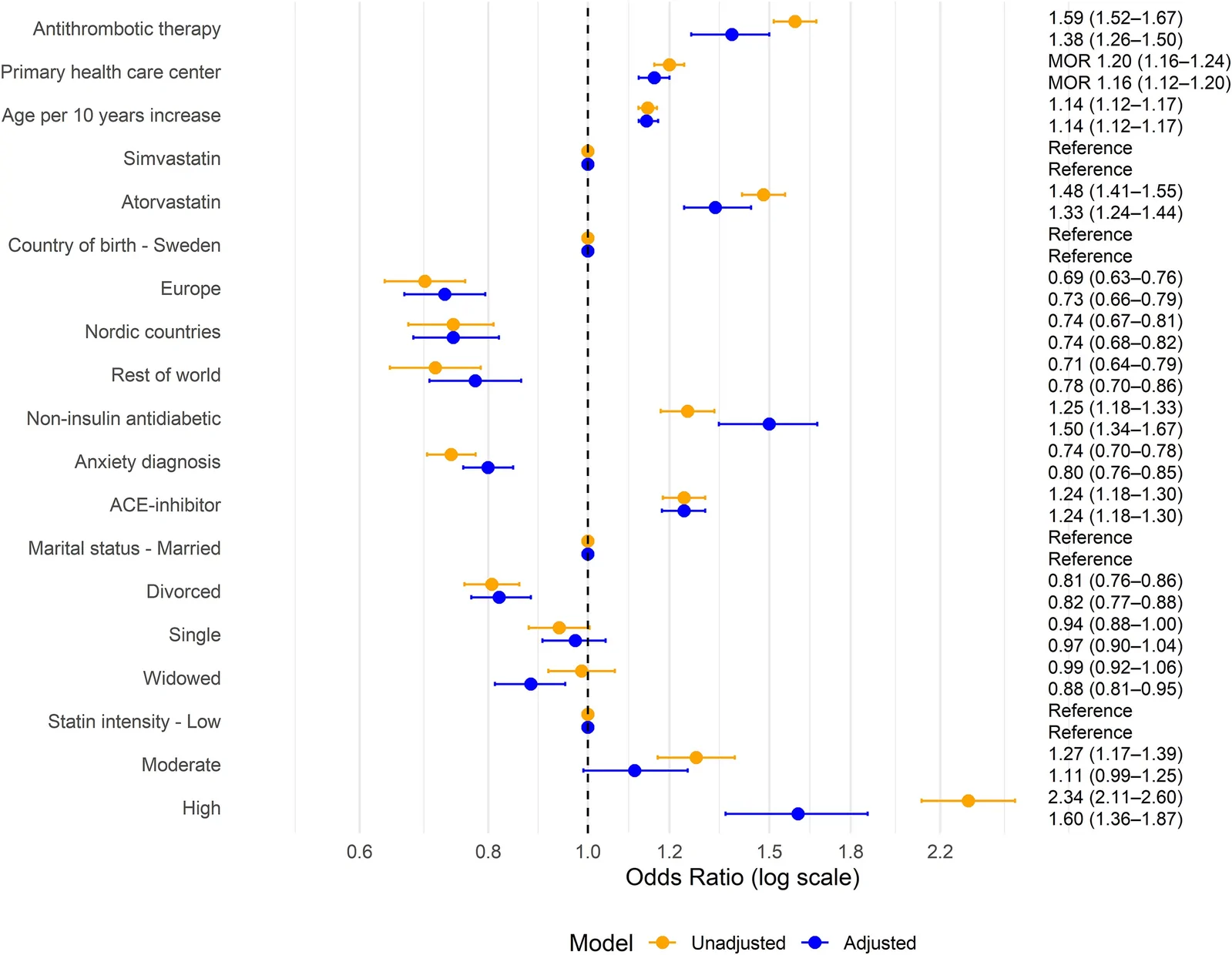
Forest plot showing effect estimates from multilevel regression models in the full study cohort for the 10 baseline characteristics most strongly associated with adherence, defined as proportion of days covered (PDC) > 80%. Odds ratios and, for primary health care centres, the median odds ratios (MORs) are presented from unadjusted and adjusted analyses, with confounder selection based on the modified disjunctive cause criterion.

The magnitudes of the associations between baseline characteristics and adherence are presented as unadjusted and adjusted estimates in *Figure 3*. The patterns of associations were similar in the unadjusted models as compared with the adjusted models, although mostly with numerically larger effect sizes in the unadjusted models. Confounder selections in the adjusted models are presented in Supplementary material online, *Figure S2*.

In the adjusted analyses, the associations between the five most influential baseline characteristics and PDC > 80% were: dispensed antithrombotic therapy (OR 1.38, 95% CI 1.26–1.50), the patient’s PHCC (MOR 1.16, 95% CI 1.12–1.20), age per 10 years increase (OR 1.14, 95% CI 1.12–1.17), initiation of atorvastatin vs. simvastatin (OR 1.33, 95% CI 1.24–1.44), and country of birth outside of Sweden [OR range between 0.73 (95% CI 0.66–0.79) and 0.78 (95% CI 0.70–0.86)]. Sensitivity analysis using PDC as a continuous variable confirmed that the top 10 baseline characteristics remained similar, except for educational status being among the top 10 most influential variables instead of statin intensity, see Supplementary material online, *Figure S3*.

### Baseline characteristics and primary preventive adherence

**Figure 4:**
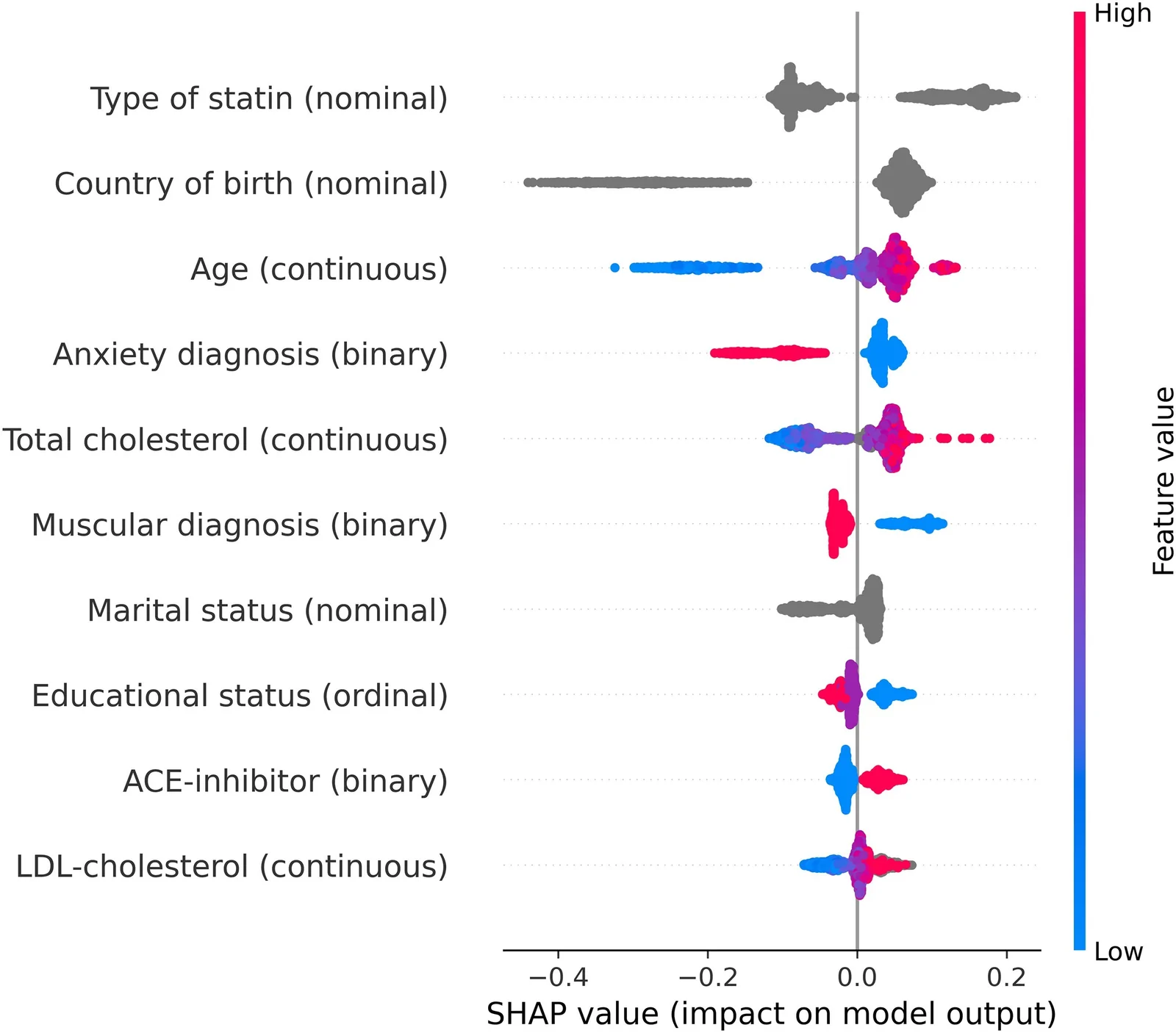
SHAP values for the primary prevention group (test data) illustrating the 10 baseline characteristics most strongly associated with predicted adherence, defined as proportion of days covered (PDC) > 80%. Each dot represents one individual. The x-axis shows how much each variable contributed to the prediction of adherence in the machine learning model. Positive SHAP values indicate higher predicted adherence, whereas negative values indicate lower predicted adherence. The colours indicate whether the value of the variable was high (red) or low (blue). ACE: angiotensin-converting enzyme. LDL: low-density lipoprotein.

**Figure 5:**
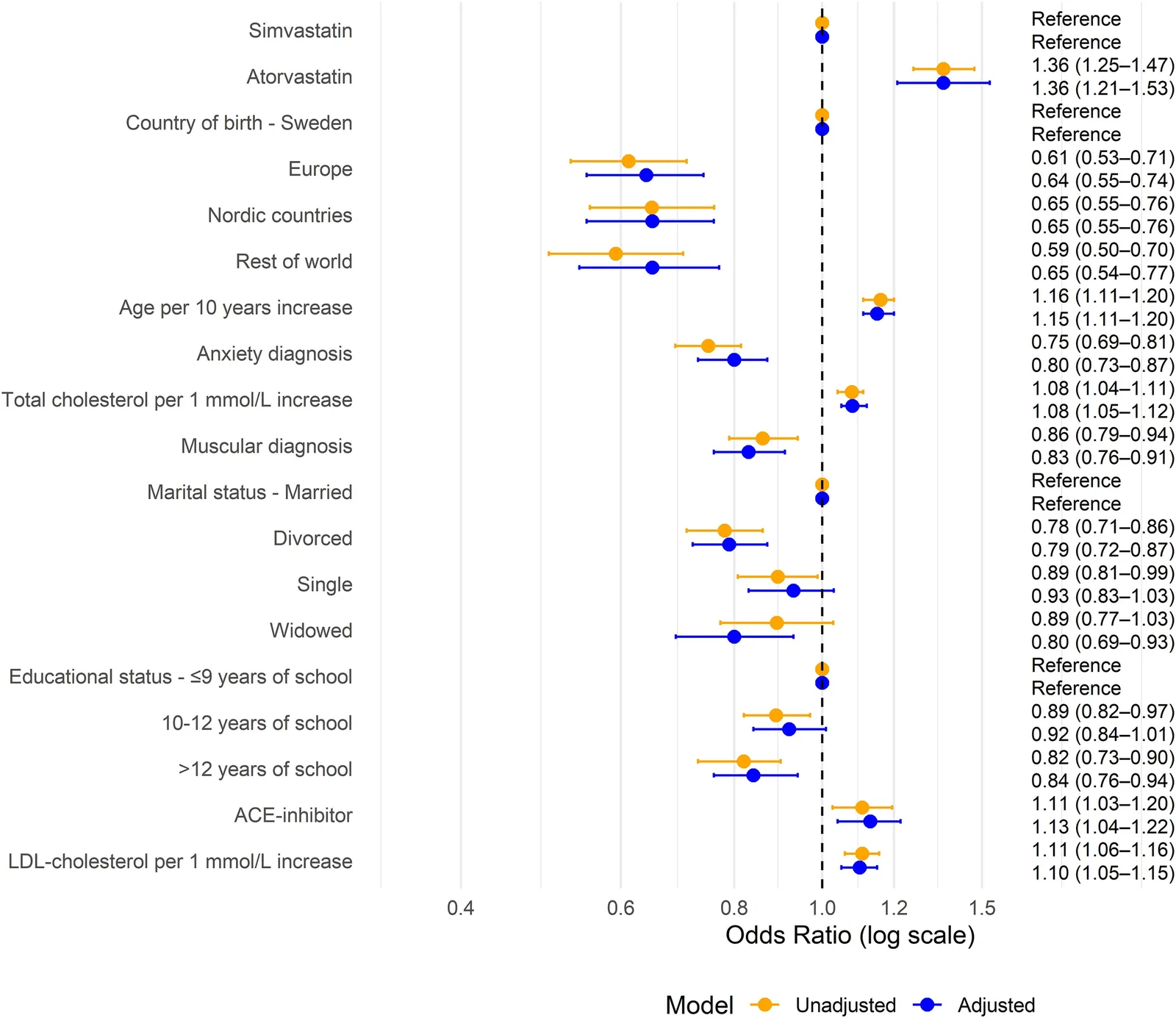
Forest plot showing effect estimates from multilevel regression models in the primary prevention group for the 10 baseline characteristics most strongly associated with adherence, defined as proportion of days covered (PDC) > 80%. Odds ratios and, for primary health care centres, the median odds ratios (MOR)s are presented from unadjusted and adjusted analyses, with confounder selection based on the modified disjunctive cause criterion.

The associations between baseline characteristics and PDC > 80% among patients in the primary prevention group are illustrated in the SHAP plot in *Figure 4*, with unadjusted and adjusted effect sizes in *Figure 5*. Confounder selections for the adjusted analyses are presented in Supplementary material online, *Figure S4*. The AUC-ROC score for the primary prevention group was 0.63 (95% CI 0.62–0.64) in the training set and 0.58 (95% CI 0.56–0.60) in the test set. In contrast to the findings when all patients were included, total cholesterol, a musculoskeletal diagnosis, educational status and LDL-C were among the most influential baseline characteristics associated with PDC > 80% in the primary prevention group. In the adjusted regression analysis, higher levels of total cholesterol and LDL-C were associated with greater adherence, OR 1.08 (95% CI 1.05–1.12) and OR 1.10 (95% CI 1.05–1.15), respectively, per 1 mmol/L increase. A musculoskeletal diagnosis at baseline was associated with decreased adherence, OR 0.83 (95% CI 0.76–0.91). Higher educational level was associated with decreased adherence, OR 0.84 (95% CI 0.76–0.94) for more than 12 years vs. 9 years or less in school.

## Discussion

In this large cohort study of 30 497 patients with hypertension in primary health care, 60.2% of patients had adequate adherence (PDC > 80%) during the 2 years following statin initiation. Adherence varied significantly across study subgroups, with the lowest adherence in the primary prevention group, higher adherence among patients with diabetes without CVD, and the highest adherence in the secondary prevention group. Explainable machine learning techniques were used to identify baseline characteristics most strongly associated with adherence. Overall, the five most important baseline characteristics were concomitant antithrombotic therapy, PHCC, age, type of statin initiated, and country of birth.

### Comparison with previous studies

Although we did not identify any studies with the same target population of hypertensive patients in a primary care setting, our results are similar to those in other populations and to a recent large systematic review and meta-analysis reporting overall adherence (PDC ≥ 80%) of 62.4%, compared with (PDC > 80%) 60.2% in our study.^19–21^ In the subgroup receiving primary preventive therapy, 54.0% achieved adequate adherence, consistent with previous primary prevention studies not limited to patients with hypertension, which have reported adherence rates ranging from 50% to 70%.^22,23^ Among patients receiving secondary preventive therapy, adherence was higher than in the primary preventive group, with 65.5% achieving PDC > 80%, in line with earlier findings in comparable populations.^24,25^

Our study included a broad range of baseline patient characteristics across multiple domains, including comorbidities, dispensed drugs, socioeconomic factors, and clinical and PHCC data. In the machine learning model, concomitant use of antithrombotic therapy, non-insulin antidiabetic drugs, and ACE inhibitors ranked among the 10 most important variables associated with higher statin adherence, whereas diagnoses of CVD and diabetes did not. A similar finding was observed in a Swedish study of patients with Type 1 diabetes, in which concomitant antihypertensive and antithrombotic treatment was associated with higher adherence to lipid-lowering therapy.^26^

Previous studies have reported higher statin adherence with increasing age, consistent with our findings.^27^ Sex was not among the 10 most influential baseline characteristics associated with adherence in our study, although prior studies have shown that men tend to be more adherent than women.^28^ Initiation of atorvastatin was associated with higher adherence compared with other statins. Few studies have explored the relationship between the type of statin and adherence. However, a Finnish primary preventive study reported that patients were more likely to adhere to rosuvastatin and fluvastatin than to simvastatin.^29^ Higher statin intensity has previously been associated with reduced adherence in primary prevention^28^ and among patients with familial hypercholesterolaemia,^30^ an effect attributed to an increased risk of side effects, particularly myalgia. Interestingly, a recent large meta-analysis of randomized clinical trials concluded that many perceived side effects are incorrectly attributed to statin therapy.^31^ In contrast, our study showed a positive association between higher statin intensity and adherence. Possible explanations are that higher intensity statins are more often dispensed to patients in the secondary prevention group, who have a stronger clinical indication and may have greater commitment and motivation to therapy after experiencing a cardiovascular event. In the adjusted regression model, the effect size of the association between PHCCs and statin adherence was modest (MOR 1.16, 95% CI 1.12–1.20). To our knowledge, variation across PHCCs within a region has not been previously examined. The present findings indicate that such variation exists and is more apparent in the SHAP analyses than in conventional regression models. These results may underscore the importance of standardized treatment guidelines, structured follow-up procedures, and educational interventions targeting PHCCs. Being born outside Sweden was associated with lower adherence, consistent with findings from a Swedish study among patients with Type 1 diabetes.^26^ Being widowed or divorced was also associated with lower adherence compared with being married, contrasting with a previous study that reported a negative association between marriage and adherence in a primary preventive context. The authors of that study found this unexpected, suggesting that spousal support would be expected to enhance adherence.^28^ Although previous studies have not demonstrated a significant association between anxiety and statin adherence, our study identified a negative association (OR 0.80, 95% CI 0.76–0.85).

### Clinical implications

The clinical benefits of statin therapy have been demonstrated in meta-analyses of randomized clinical trials,^3^ as well as in observational studies.^32^ The data-driven machine learning approach applied in this study to explore associations between patient characteristics and statin adherence adds to the existing literature. Several groups were identified as having a higher likelihood of suboptimal adherence, including patients born outside Sweden, unmarried patients, younger patients, and patients with anxiety. These findings may help identify populations that could potentially benefit from adherence-support interventions, while acknowledging that the observed associations are not causal. The observed variability in adherence across PHCCs indicates a need for more standardized treatment guidelines and structured follow-up procedures. Reducing inter-centre variability may contribute to more consistent and equitable care for patients prescribed statin therapy. Not all patients diagnosed with CVD fill prescriptions for guideline-recommended cardiovascular drugs.^33^ This may partly explain why the use of other cardiovascular drugs, rather than CVD diagnoses *per se*, was associated with statin adherence in the present study.

### Strengths and limitations

The study has several strengths. It included a large region-wide cohort of patients with hypertension from all PHCCs, minimizing selection bias and enhancing the representativeness of routine primary health care. Data on comorbidities, drugs, and socioeconomic factors were obtained from high-quality national and regional registers. Explainable machine learning was used to select and rank patient characteristics most important for the prediction of adherence, without requiring specification of potentially complex interactions and functional forms. Subsequently, generalized linear mixed models were used to obtain interpretable estimates of total effects. This sequential approach may have missed variables with a large proportion of their effect mediated by other variables included in the machine learning model. However, similar risks exist in classical variable selection approaches such as forward selection. Finally, the use of comprehensive baseline data to classify statin indication reduces the risk of misclassification.

The study also has limitations. Adherence estimated from dispensed prescriptions may overestimate actual medication intake, as dispensing does not necessarily equate to consumption. Adherence to statin therapy may also be overestimated, as patients who were prescribed statins but never dispensed a prescription were not captured, and the interval between prescription and first dispensation was unavailable. Some potentially relevant baseline characteristics were lacking, such as family history of CVD and patient-related factors including health literacy, beliefs, and attitudes towards preventive medication, as well as prescriber- and PHCC-level organizational factors. Consequently, SHAP-based rankings may change if additional predictors are included. In addition, mixed model estimates of total effects can be interpreted causally only if the covariate adjustment set is correct. The moderate AUC values of the XGBoost model underscore the need for additional data to better capture the complexity of adherence behaviour. However, the study contained an extensive list of routinely collected primary health care data, and some previous research suggests that feature-importance rankings may remain informative despite modest model discrimination when sample size is sufficiently large, although the result should still be interpreted cautiously as exploratory rather than definitive findings.^34^ Also, prediction was not the primary aim of this study. Rather, the objective was to explore and rank the relative importance of available baseline variables. Qualitative research, such as interviews with medical staff at PHCCs with high adherence to identify facilitating factors, as well as interviews with patients initiating statin therapy, could help elucidate important determinants of adherence that are not captured in register data. The two-step approach using XGBoost and SHAP followed by regression analysis may have introduced data-driven selection bias; however, it also enabled the identification of predictors for which prior hypotheses were limited. Although XGBoost can handle missing values natively, substantial missingness in some clinical variables, such as HbA1c and waist circumference, may have reduced their importance in the SHAP analysis and may have introduced bias if not missing completely at random. Finally, the use of data only up to 2017 represents an important limitation, as the findings should be interpreted in the context of the clinical guidelines and prescribing patterns prevailing during the period of study inclusion between 2012 and 2015,^13^ before the introduction of lower lipid targets and the more widespread use of atorvastatin, rosuvastatin, and combination therapy with ezetimibe.^35^ However, we believe several patient baseline characteristics, including age, socioeconomic characteristics, country of birth, comorbidity, and variation between primary health care centres, are likely to remain relevant to statin adherence also in a contemporary primary health care setting. As our study found higher adherence among patients treated with atorvastatin compared with simvastatin, current adherence may differ from that observed in our cohort and may potentially be more favourable.

## Conclusion

In this large registry-based cohort of patients with hypertension in Swedish primary care, only 60% achieved adequate statin adherence during the 2 years after initiation. Adherence differed across indications, being lowest in primary prevention and highest in secondary prevention. In the overall cohort, adherence was most strongly associated with concomitant cardiometabolic medication use, age, statin type, and socioeconomic factors, and varied across PHCCs within the region. These findings indicate that suboptimal adherence represents a missed opportunity for effective cardiovascular prevention and suggest that targeted support and structured follow-up may be needed to improve equitable implementation of preventive statin therapy.

## Supplementary Material

pvag048_Supplementary_Data

## Data Availability

The data on which this study is based cannot be shared publicly due to legal and ethical regulations set by the Swedish authorities. Data can, however, after reasonable request, be made available to researchers who meet the criteria for access to confidential data after permission from Region Västra Götaland, the Swedish National Board of Health and Welfare, and Statistics Sweden.
