# Supplementary material for: Statin adherence among patients with hypertension in Swedish primary health care: a QregPV cohort study using explainable machine learning
Source: Eur Heart J Cardiovasc Pharmacother. 2026 Jun 29;12(6):458–67. doi: 10.1093/ehjcvp/pvag048 (PMC13594359; doi:10.1093/ehjcvp/pvag048)
Supplement: Supplementary file 1 [file pvag048_supplementary_data.pdf]

# Supplemental Material

This supplemental material has been provided by the authors to give readers additional information about their work.

Supplement to:

## Statin adherence among patients with hypertension in Swedish primary health care: a QregPV cohort study using explainable machine learning

Johan Sandslätt, Kristina Bengtsson Boström, Maria Åberg, Johan-Emil Bager, Koen Simons, Georgios Mourtzinis, Per Hjerpe, Tobias Andersson

## Data sources

The Prescribed Drug Register (2008–2017) provided data on all dispensed medications including whether statins were prescribed from primary or secondary health care. Inpatient (1997–2017) and outpatient hospital diagnoses (2001–2017) were obtained from the National Patient Register.[1] The regional primary-care database in Region Västra Götaland (VEGA) contributed with information on primary and hospital health care visits in VGR (2000–2017). Sociodemographic variables, including country of birth, disposable income, educational level, and marital status, were retrieved from the Longitudinal Integrated Database for Health Insurance and Labour Market Studies (LISA) register (2000–2017).[2] Mortality data, used to define patients' observation periods, were obtained from the Cause of Death Register (data up to 2017).[3] Clinical diagnoses were coded according to the International Classification of Diseases, 10th Revision (ICD-10), and medications were classified using the Anatomical Therapeutic Chemical (ATC) classification system.

## Statistical analysis

In brief, the machine learning approach was used as an exploratory prediction method to rank baseline characteristics according to their contribution to adherence prediction. XGBoost can capture complex and non-linear relationships between variables, while SHAP values provide a way to visualise and rank how much each variable contributed to the model predictions. By definition, this ranking is relative to all other variables included in the model. More importantly, the ranking ignores variables not included in the model. For instance, assume two variables T and F are correlated with one another. Assume T is a true predict and F does not independently predict the outcome. Using SHAP to rank variables, we expect F to have a low rank in models

that include both T and F as candidate variables, however, F may obtain a high rank if T is not considered as a candidate variable. Using all available variables a priori considered potentially relevant, these analyses were used to select variables for further examination in regression models and should not be interpreted as causal analyses.

To assess out-of-sample predictive performance, the dataset was randomly split into 80% for model training and 20% for testing. The XGBoost model natively handled categorical data and missing data without requiring prior imputation and was optimized through Bayesian hyperparameter tuning using the Optuna framework.[4] Specifically, five hundred optimization trials were performed with 5-fold cross-validation, using the area under the receiver operating characteristic curve (AUC-ROC) as the performance metric. Although model prediction was not the primary aim of the study, calibration plots were generated in the test data for descriptive purposes. The final optimized model was fitted to the entire training data. Model interpretability was assessed using SHAP values from the final optimized model applied to the test data, and the ten variables with the highest mean absolute SHAP values were visualised to illustrate their relative contribution to adherence. The machine learning analyses were performed using the Python implementations of XGBoost and SHAP. Random seed 42 was used for the 80/20 train-test split and the XGBoost model. The multilevel logistic regression models were adjusted using the modified disjunctive cause criterion, which entails adjusting for variables that are causes of the exposure, the outcome, or both, while excluding mediators that lie on the causal pathway between exposure and outcome, as well as instrumental variables that affect the exposure but have no direct effect on the outcome. The disjunctive cause criterion is based on causal principles and aims to estimate total effects, whereas XGBoost and SHAP ignore concepts such as mediation.

**Supplementary Table S1.** Baseline characteristics of individuals included in the study including socioeconomic variables, comorbidities and filled prescriptions within 12 months.

| | Overall | Primary prevention | Diabetes without CVD | Secondary prevention | Missing (%) |
|---|---|---|---|---|---|
| Number of individuals | 30 497 | 12 136 | 5073 | 13 288 | |
| Age, years (SD) | 66.3 (11.5) | 62.5 (10.2) | 62.5 (10.5) | 71.2 (11.0) | 0.0 |
| Women, n (%) | 14 565 (47.8) | 6330 (52.2) | 2189 (43.2) | 6046 (45.5) | 0.0 |
| Weight, kg (SD) | 84.4 (18.5) | 83.4 (17.6) | 92.7 (19.7) | 81.7 (17.8) | 14.9 |
| Height, cm (SD) | 170.5 (9.8) | 170.7 (9.6) | 171.6 (9.9) | 169.9 (9.9) | 17.0 |
| BMI, kg/m$^2$ (SD) | 29.0 (5.3) | 28.5 (4.9) | 31.4 (5.8) | 28.3 (5.2) | 19.1 |
| Waist circumference, cm (SD) | 101.1 (13.6) | 99.1 (13.0) | 107.1 (13.7) | 100.1 (13.3) | 27.2 |
| Total cholesterol, mmol/L (SD) | 6.1 (1.3) | 6.6 (1.2) | 5.6 (1.2) | 5.7 (1.2) | 16.5 |
| LDL-cholesterol, mmol/L (SD) | 4.1 (1.0) | 4.5 (0.9) | 3.8 (0.8) | 3.8 (0.9) | 23.3 |
| Triglycerides, mmol/L (SD) | 1.8 (1.2) | 1.9 (1.2) | 2.0 (1.3) | 1.7 (1.0) | 22.8 |
| SBP, mmHg (SD) | 140.3 (17.9) | 141.7 (17.5) | 136.8 (16.8) | 140.4 (18.5) | 4.7 |
| DBP, mmHg (SD) | 82.8 (12.3) | 85.7 (11.9) | 81.5 (12.0) | 80.6 (12.3) | 4.8 |
| HbA1c, mmol/mol (SD) | 49.4 (15.1) | 38.0 (4.7) | 53.0 (15.6) | 49.4 (14.9) | 68.5* |
| Smoker, n (%) | 4403 (17.8) | 1897 (18.9) | 756 (16.0) | 1750 (17.5) | 18.7 |
| Income, median, 1000 SEK (IQR) | 175.3 (131.1–260.2) | 205.9 (140.6–285.8) | 183.7 (131.8–266.0) | 155.6 (125.0–226.6) | 0.1 |
| Educational status, n (%) | | | | | 1.8 |
| >12 years in school | 6489 (21.7) | 2981 (24.8) | 1016 (20.5) | 2492 (19.2) | |
| 10–12 years in school | 13 106 (43.8) | 5572 (46.4) | 2286 (46.1) | 5248 (40.4) | |
| 0–9 years in school | 10 358 (34.6) | 3456 (28.8) | 1653 (33.4) | 5249 (40.4) | |
| Marital status, n (%) | | | | | 0.1 |
| Married | 16 621 (54.5) | 7036 (58.0) | 2699 (53.2) | 6886 (51.9) | |
| Divorced | 5714 (18.8) | 2246 (18.5) | 957 (18.9) | 2511 (18.9) | |
| Single | 4519 (14.8) | 1983 (16.4) | 1022 (20.2) | 1514 (11.4) | |
| Widowed | 3619 (11.9) | 862 (7.1) | 391 (7.7) | 2366 (17.8) | |
| Country of birth, n (%) | | | | | 0.0 |
| Sweden | 24 614 (80.7) | 10 090 (82.5) | 3937 (77.4) | 10 587 (80.4) | |
| Europe | 2219 (7.3) | 825 (6.8) | 438 (8.6) | 956 (7.2) | |
| Nordic countries | 1888 (6.2) | 682 (5.6) | 247 (4.9) | 959 (7.2) | |
| Rest of the world | 1775 (5.8) | 616 (5.1) | 459 (9.0) | 700 (5.3) | |
| Baseline comorbidity, n (%) | | | | | 0.0 |
| Cancer (C00–C97) | 4904 (16.1) | 1557 (12.8) | 628 (12.4) | 2719 (20.5) | |
| Hypothyroidism (E03) | 2400 (7.9) | 852 (7.0) | 354 (7.0) | 1194 (9.0) | |

| | | | | | |
|---|---|---|---|---|---|
| Hyperthyroidism (E05) | 416 (1.4) | 162 (1.3) | 64 (1.3) | 190 (1.4) | |
| Diabetes mellitus (E10–E14) | 7998 (26.2) | 0 (0.0) | 5073 (100.0) | 2925 (22.0) | |
| Obesity (E66) | 6185 (20.3) | 1981 (16.3) | 2054 (40.5) | 2150 (16.2) | |
| Dementia (F00–F03, G30) | 455 (1.5) | 48 (0.4) | 21 (0.4) | 386 (2.9) | |
| Psychosis (F20–F29) | 355 (1.2) | 107 (0.9) | 101 (2.0) | 147 (1.1) | |
| Mood disorder (F30–F39) | 5944 (19.5) | 2267 (18.7) | 959 (18.9) | 2718 (20.5) | |
| Anxiety disorder (F40–F48) | 7113 (23.3) | 3033 (25.0) | 1167 (23.0) | 2913 (21.9) | |
| TIA (G45) | 1024 (3.4) | 0 (0.0) | 0 (0.0) | 1024 (7.7) | |
| IHD (I20–I25) | 4700 (15.4) | 0 (0.0) | 0 (0.0) | 4700 (35.4) | |
| MI (I21–I22) | 1554 (5.1) | 0 (0.0) | 0 (0.0) | 1554 (11.7) | |
| Atrial fibrillation/flutter (I48) | 2787 (9.1) | 625 (5.1) | 277 (5.5) | 1885 (14.2) | |
| Heart failure (I50) | 1824 (6.0) | 255 (2.1) | 165 (3.3) | 1404 (10.6) | |
| Subarachnoid bleeding (I60) | 113 (0.4) | 23 (0.2) | 2 (0.0) | 88 (0.7) | |
| Cerebral bleeding (I61+I629) | 260 (0.9) | 33 (0.3) | 11 (0.2) | 216 (1.6) | |
| Cerebral infarction (I63) | 1920 (6.3) | 0 (0.0) | 0 (0.0) | 1920 (14.4) | |
| Cerebrovascular disease, unspecified (I64 + I679) | 955 (3.1) | 0 (0.0) | 0 (0.0) | 955 (7.2) | |
| Sequelae of cerebrovascular (I69) | 922 (3.0) | 0 (0.0) | 0 (0.0) | 922 (6.9) | |
| Atherosclerosis (I70) | 530 (1.7) | 0 (0.0) | 0 (0.0) | 530 (4.0) | |
| Aneurysm (I71, I72, I74) | 659 (2.2) | 142 (1.2) | 28 (0.6) | 489 (3.7) | |
| PAD (I739) | 850 (2.8) | 0 (0.0) | 0 (0.0) | 850 (6.4) | |
| COPD (J44) | 2171 (7.1) | 583 (4.8) | 263 (5.2) | 1325 (10.0) | |
| Asthma (J45) | 2408 (7.9) | 859 (7.1) | 438 (8.6) | 1111 (8.4) | |
| Liver disease (K70–K77) | 245 (0.8) | 52 (0.4) | 61 (1.2) | 132 (1.0) | |
| Musculoskeletal disorder (M00–M79) | 23 958 (78.6) | 9233 (76.1) | 3883 (76.5) | 11 842 (81.6) | |
| Rheumatoid arthritis (M05–M06) | 820 (2.7) | 299 (2.5) | 117 (2.3) | 404 (3.0) | |
| Kidney failure (N18–N19) | 750 (2.5) | 183 (1.5) | 91 (1.8) | 476 (3.6) | |
| Filled prescription ≤1 year, n (%) | | | | | 0.0 |
| Insulin | 1582 (5.2) | 0 (0.0) | 788 (15.5) | 794 (6.0) | |
| Other glucose lowering drug | 5837 (19.1) | 0 (0.0) | 3949 (77.8) | 1888 (14.2) | |
| Fibrate | 145 (0.5) | 33 (0.3) | 27 (0.5) | 85 (0.6) | |
| Resins | 81 (0.3) | 24 (0.2) | 16 (0.3) | 41 (0.3) | |
| Ezetimibe | 73 (0.2) | 12 (0.1) | 13 (0.3) | 48 (0.4) | |
| Beta-blocker | 13 508 (44.3) | 3914 (32.3) | 1681 (33.1) | 7913 (59.5) | |

| | | | | | |
|---|---|---|---|---|---|
| ACE inhibitor | 12 445 (40.8) | 4335 (35.7) | 2364 (46.6) | 6746 (43.2) | |
| Angiotensin blocker | 10 024 (32.9) | 4000 (33.0) | 1771 (34.9) | 4250 (32.0) | |
| Calcium channel blocker | 12 693 (41.6) | 4744 (39.1) | 1948 (38.4) | 6001 (45.2) | |
| Organic nitrates | 3742 (12.3) | 0 (0.0) | 0 (0.0) | 3742 (28.2) | |
| Thiazide diuretic | 9465 (31.0) | 3643 (30.0) | 1675 (33.0) | 4150 (31.2) | |
| Alpha-blocker | 313 (1.0) | 88 (0.7) | 48 (0.9) | 177 (1.3) | |
| Aldosterone-blocker | 946 (3.1) | 180 (1.5) | 126 (2.5) | 640 (4.8) | |
| Loop diuretics | 3299 (10.8) | 619 (5.1) | 458 (9.0) | 2222 (16.7) | |
| Antithrombotic therapy | 13 254 (43.5) | 869 (7.2) | 337 (6.6) | 12 048 (90.7) | |
| Heparin | 784 (2.6) | 217 (1.8) | 86 (1.7) | 481 (3.6) | |
| NOAK | 626 (2.1) | 178 (1.5) | 47 (0.9) | 401 (3.0) | |
| Warfarin | 2021 (6.6) | 596 (4.9) | 247 (4.9) | 1178 (8.9) | |
| Acetylsalicylic acid | 9427 (30.9) | 0 (0.0) | 0 (0.0) | 9427 (70.9) | |
| Clopidogrel | 3193 (10.5) | 0 (0.0) | 0 (0.0) | 3193 (24.0) | |

Categorical variables are presented as number (%) and continuous variables as mean (SD) unless stated otherwise. Diagnoses are coded according to the International Statistical Classification of Diseases and Related Health Problems 10th revision (ICD-10). CVD: cardiovascular disease. SD: standard deviation, SBP: systolic blood pressure, DBP: diastolic blood pressure, COPD: chronic obstructive pulmonary disease, TIA: transient ischaemic attack, IHD: ischaemic heart disease, MI: myocardial infarction, PAD: peripheral artery disease, ACE: angiotensin-converting enzyme, NOAK: non-vitamin K oral anticoagulant.
*10% missing in the diabetes without CVD group.

**Supplementary Table S2.** Filled prescriptions of statins.

| Filled prescription of statin | Overall | Primary prevention | Diabetes without CVD | Secondary prevention |
|---|---|---|---|---|
| Simvastatin | 17 604 (57.7) | 7744 (63.8) | 3353 (66.1) | 6507 (49.0) |
| Atorvastatin | 12 698 (41.6) | 4318 (35.6) | 1700 (33.5) | 6680 (50.3) |
| Rosuvastatin | 146 (0.5) | 61 (0.5) | 14 (0.3) | 71 (0.5) |
| Pravastatin/Fluvastatin | 49 (0.2) | 13 (0.1) | 6 (0.1) | 30 (0.2) |
| First filled prescription in 2-year period | | | | |
| Primary care | 20 929 (68.6) | 11 180 (92.1) | 4749 (93.6) | 5000 (37.6) |
| Secondary care | 9568 (31.4) | 956 (7.9) | 324 (6.4) | 8288 (62.4) |
| Last filled prescription in 2-year period | | | | |
| Primary care | 26 960 (88.4) | 11 369 (93.7) | 4774 (94.1) | 10 817 (81.4) |
| Secondary care | 3537 (11.6) | 767 (6.3) | 299 (5.9) | 2471 (18.6) |

Data are presented as numbers (%). CVD: cardiovascular disease.

**Supplementary Table S3.** Adherence (PDC) during a 2-year period after initiation of statin therapy.

| | **Overall** | **Primary prevention** | **Diabetes without CVD** | **Secondary prevention** | **P-value** |
|---|---|---|---|---|---|
| PDC, % (SD) | 73.2 (31.9) | 68.6 (33.0) | 74.6 (30.3) | 76.7 (31.0) | P < 0.001 |
| PDC category, n (%) | | | | | |
| 0 to 20% | 3909 (12.8) | 1950 (16.1) | 528 (10.4) | 1431 (10.8) | - |
| >20 to 40% | 2364 (7.8) | 1041 (8.6) | 363 (7.2) | 960 (7.2) | - |
| >40 to 60% | 3105 (10.2) | 1433 (11.8) | 558 (11.0) | 1114 (8.4) | - |
| >60 to 80% | 2752 (9.0) | 1156 (9.5) | 512 (10.1) | 1084 (8.2) | - |
| >80 to 100% | 18 367 (60.2) | 6556 (54.0) | 3112 (61.3) | 8699 (65.5) | P < 0.001 |

PDC: proportion of days covered, CVD: cardiovascular disease.

**Supplementary Table S4.** LDL cholesterol levels at baseline and at 1 and 2 years after initiation of statin therapy, according to adherence status.

| | PDC category | LDL baseline mean (SD) mmol/L | n | LDL year 1 mean (SD) mmol/L | n | LDL year 2 mean (SD) mmol/L | n | LDL-change between baseline and year 2 mean (SD) mmol/L | P-value for change in LDL |
|---|---|---|---|---|---|---|---|---|---|
| Overall cohort | 0 to 20% | 4.12 (0.98) | 3133 | 3.63 (1.02) | 1793 | 3.9 (0.93) | 1641 | -0.19 (0.68) | <0.001 |
| | >20 to 40% | 4.10 (0.96) | 1829 | 3.38 (1.11) | 1222 | 3.76 (1.05) | 1132 | -0.31 (0.88) | <0.001 |
| | >40 to 60% | 4.11 (0.99) | 2449 | 2.98 (1.04) | 1749 | 3.43 (1.11) | 1647 | -0.68 (1.03) | <0.001 |
| | >60 to 80% | 4.09 (1.01) | 2170 | 2.81 (1.08) | 1613 | 2.83 (1.01) | 1549 | -1.28 (1.02) | <0.001 |
| | >80 to 100% | 4.07 (0.99) | 13 821 | 2.35 (0.75) | 10 555 | 2.35 (0.76) | 9771 | -1.73 (0.82) | <0.001 |
| Primary prevention group | 0 to 20% | 4.44 (0.93) | 1656 | 3.87 (1.04) | 874 | 4.18 (0.91) | 810 | -0.25 (0.71) | <0.001 |
| | >20 to 40% | 4.43 (0.93) | 858 | 3.63 (1.14) | 545 | 4.05 (1.06) | 497 | -0.38 (0.90) | <0.001 |
| | >40 to 60% | 4.48 (0.94) | 1187 | 3.19 (1.08) | 827 | 3.68 (1.08) | 762 | -0.77 (1.04) | <0.001 |
| | >60 to 80% | 4.54 (0.97) | 966 | 3.15 (1.10) | 703 | 3.13 (1.04) | 660 | -1.44 (1.07) | <0.001 |
| | >80 to 100% | 4.56 (0.92) | 5413 | 2.69 (0.73) | 4120 | 2.69 (0.76) | 3633 | -1.87 (0.84) | <0.001 |
| Diabetes without CVD | 0 to 20% | 3.71 (0.85) | 491 | 3.41 (0.89) | 307 | 3.58 (0.83) | 317 | -0.13 (0.63) | <0.001 |
| | >20 to 40% | 3.77 (0.77) | 318 | 3.24 (0.94) | 221 | 3.51 (0.92) | 237 | -0.26 (0.82) | <0.001 |
| | >40 to 60% | 3.75 (0.79) | 499 | 2.82 (0.92) | 372 | 3.15 (1.03) | 364 | -0.64 (0.95) | <0.001 |
| | >60 to 80% | 3.72 (0.78) | 462 | 2.58 (0.92) | 356 | 2.52 (0.81) | 377 | -1.17 (0.90) | <0.001 |
| | >80 to 100% | 3.77 (0.84) | 2796 | 2.21 (0.67) | 2141 | 2.23 (0.66) | 2121 | -1.57 (0.76) | <0.001 |
| Secondary prevention group | 0 to 20% | 3.79 (0.92) | 986 | 3.40 (0.98) | 612 | 3.66 (0.90) | 514 | -0.13 (0.66) | <0.001 |
| | >20 to 40% | 3.83 (0.94) | 653 | 3.16 (1.10) | 456 | 3.54 (1.02) | 398 | -0.26 (0.89) | <0.001 |
| | >40 to 60% | 3.78 (0.97) | 763 | 2.78 (0.99) | 550 | 3.27 (1.11) | 521 | -0.59 (1.05) | <0.001 |
| | >60 to 80% | 3.75 (0.96) | 742 | 2.54 (1.03) | 554 | 2.68 (0.99) | 512 | -1.13 (1.01) | <0.001 |
| | >80 to 100% | 3.75 (0.92) | 5612 | 2.09 (0.67) | 4294 | 2.10 (0.68) | 4017 | -1.67 (0.80) | <0.001 |

P-values were calculated using paired t-tests. LDL: low-density lipoprotein, PDC: proportion of days covered, SD: standard deviation.

**Supplementary Table S5.** Proportion of patients achieving LDL-C targets, according to adherence status.

| | PDC category | LDL baseline <3.0 % | LDL year 1 <3.0 % | LDL year 2 <3.0 % | LDL baseline <2.6 % | LDL year 1 <2.6 % | LDL year 2 <2.6 % | LDL baseline <1.8 % | LDL year 1 <1.8 % | LDL year 2 <1.8 % |
|---|---|---|---|---|---|---|---|---|---|---|
| Overall cohort | 0 to 20% | 9.8 | 25.0 | 15.1 | 4.0 | 14.1 | 6.0 | 0.6 | 3.2 | 0.9 |
| | >20 to 40% | 10.2 | 37.4 | 22.9 | 4.7 | 23.7 | 13.1 | 0.8 | 6.8 | 2.2 |
| | >40 to 60% | 10.2 | 55.1 | 35.5 | 4.2 | 38.1 | 23.4 | 0.9 | 8.3 | 5.1 |
| | >60 to 80% | 11.0 | 61.8 | 60.1 | 4.2 | 48.2 | 44.6 | 1.0 | 13.9 | 12.7 |
| | >80 to 100% | 11.4 | 82.1 | 82.2 | 5.0 | 65.6 | 66.2 | 0.6 | 20.3 | 21.0 |
| Primary prevention group | 0 to 20% | 3.6 | 18.5 | 7.7 | 1.6 | 10.5 | 2.8 | 0.3 | 2.2 | 0.6 |
| | >20 to 40% | 4.2 | 30.6 | 15.3 | 1.9 | 18.2 | 8.5 | 0.5 | 4.4 | 2.2 |
| | >40 to 60% | 4.0 | 48.0 | 26.8 | 1.6 | 30.5 | 16.1 | 0.4 | 5.4 | 2.2 |
| | >60 to 80% | 4.8 | 50.2 | 49.5 | 1.6 | 34.0 | 32.7 | 0.4 | 6.4 | 4.8 |
| | >80 to 100% | 3.2 | 70.0 | 69.5 | 1.3 | 45.9 | 46.4 | 0.2 | 6.8 | 7.0 |
| Diabetes without CVD | 0 to 20% | 16.1 | 30.6 | 19.9 | 6.3 | 16.6 | 10.1 | 0.8 | 3.6 | 1.3 |
| | >20 to 40% | 12.3 | 39.4 | 26.2 | 5.0 | 25.3 | 16.9 | 0.9 | 4.5 | 1.3 |
| | >40 to 60% | 11.0 | 61.8 | 44.2 | 3.4 | 44.1 | 32.4 | 1.0 | 9.1 | 7.1 |
| | >60 to 80% | 13.2 | 70.5 | 73.2 | 5.0 | 57.3 | 56.5 | 0.4 | 15.7 | 16.4 |
| | >80 to 100% | 14.1 | 88.6 | 88.0 | 4.8 | 75.8 | 74.4 | 0.5 | 22.4 | 23.3 |
| Secondary prevention group | 0 to 20% | 17.2 | 31.5 | 23.7 | 6.9 | 17.8 | 8.4 | 1.0 | 4.4 | 1.0 |
| | >20 to 40% | 17.0 | 44.5 | 30.4 | 8.3 | 29.6 | 16.6 | 1.1 | 10.7 | 2.8 |
| | >40 to 60% | 19.5 | 61.1 | 42.0 | 8.7 | 45.6 | 27.6 | 1.4 | 12.2 | 7.9 |
| | >60 to 80% | 17.7 | 70.9 | 64.1 | 7.3 | 60.5 | 51.2 | 2.2 | 22.2 | 19.9 |
| | >80 to 100% | 17.9 | 90.5 | 90.8 | 8.6 | 79.4 | 79.8 | 1.1 | 32.3 | 32.4 |

LDL-C: low-density lipoprotein cholesterol, PDC: proportion of days covered.

**Supplementary Table S6.** Adherence calculated with the MPR-like measure CMA2 during a 2-year period after initiation of statin therapy.

| | Overall | Primary prevention | Diabetes without CVD | Secondary prevention | P-value |
|---|---|---|---|---|---|
| CMA2, % mean (SD) | 84.8 (75.7) | 77.2 (54.5) | 84.1 (37.1) | 92.0 (99.1) | P < 0.001 |
| CMA2 category, n (%) | | | | | |
| 0 to 20% | 3764 (12.3) | 1882 (15.5) | 498 (9.8) | 1384 (10.4) | - |
| >20 to 40% | 2126 (7.0) | 933 (7.7) | 315 (6.2) | 878 (6.6) | - |
| >40 to 60% | 3025 (9.9) | 1393 (11.5) | 545 (10.7) | 1087 (8.2) | - |
| >60 to 80% | 1777 (5.8) | 746 (6.1) | 340 (6.7) | 691 (5.2) | - |
| >80% | 19 805 (64.9) | 7182 (59.2) | 3375 (66.5) | 9248 (69.6) | P < 0.001 |

CMA2: Continuous multiple-interval measures of medication availability/gaps version 2, corresponding to the medication possession ratio (MPR).

**Supplementary Table S7.** Hyperparameter search ranges and final optimised values for the XGBoost models in the full study cohort and primary prevention cohort.

| Hyperparameter | Search range | Value – full study cohort | Value – primary prevention cohort |
|---|---|---|---|
| learning_rate | 0.01–0.05 | 0.04742359179891845 | 0.04051088855002196 |
| max_depth | 2–4 | 2 | 3 |
| subsample | 0.6–1.0 | 0.9156086136045007 | 0.9554758602508218 |
| colsample_bytree | 0.6–1.0 | 0.9864735636744846 | 0.8646574528948562 |
| min_child_weight | 2–20 | 15.905631792118568 | 3.211859435105091 |
| gamma | 0.0–5.0 | 0.012181272005722077 | 1.3642761549547824 |
| reg_alpha | 1.0–20.0 | 5.604105325875589 | 11.25308864935976 |
| reg_lambda | 1.0–20.0 | 3.483526790401456 | 12.973737943869208 |
| n_estimators * | 400–1200 | 789 | 1012 |

* For n_estimators, the Optuna-selected value represents the maximum number of boosting rounds. After early stopping during cross validation, the final models used 528 boosting rounds in the full cohort and 83 in the primary prevention cohort.

**A**

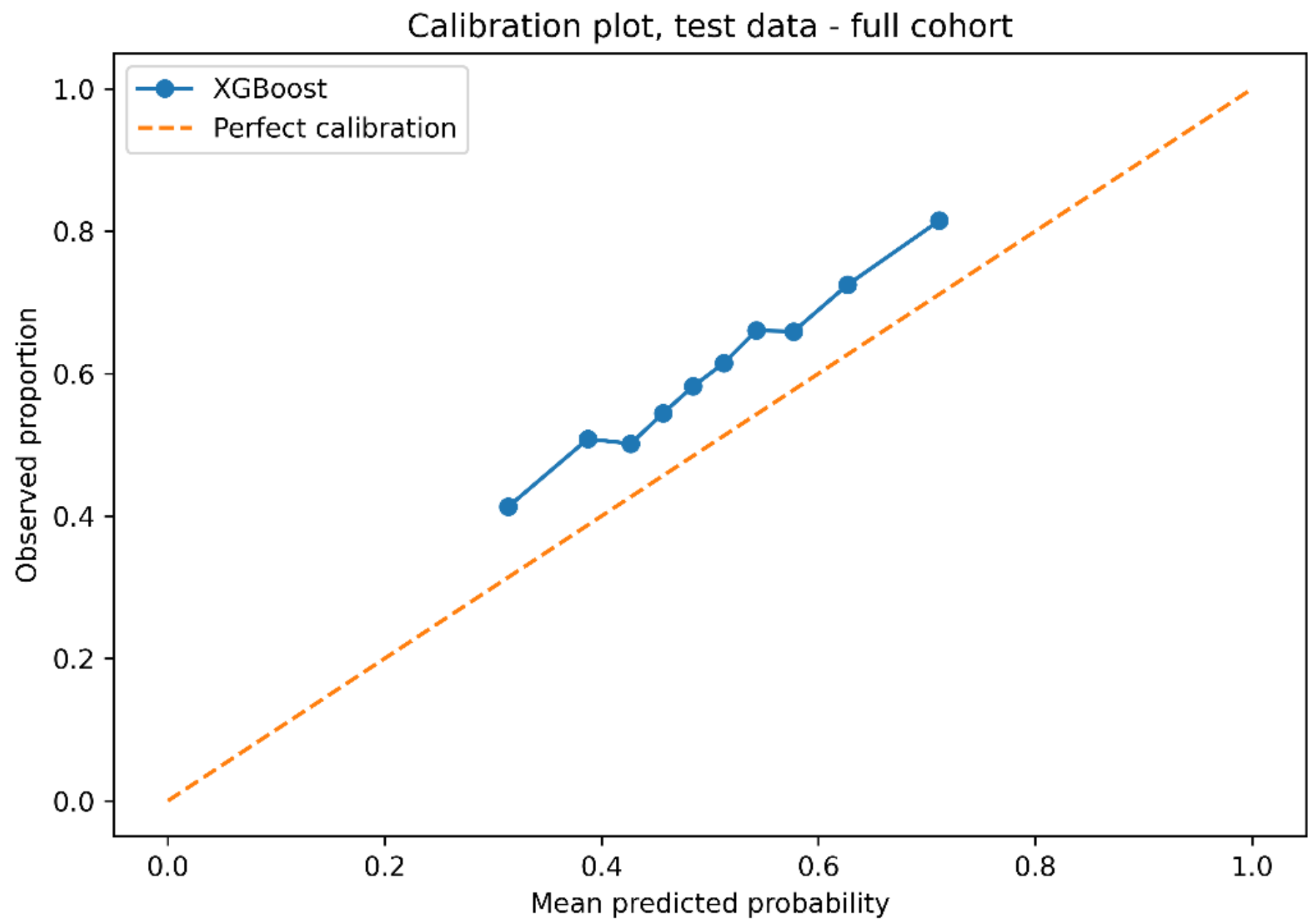


**B**

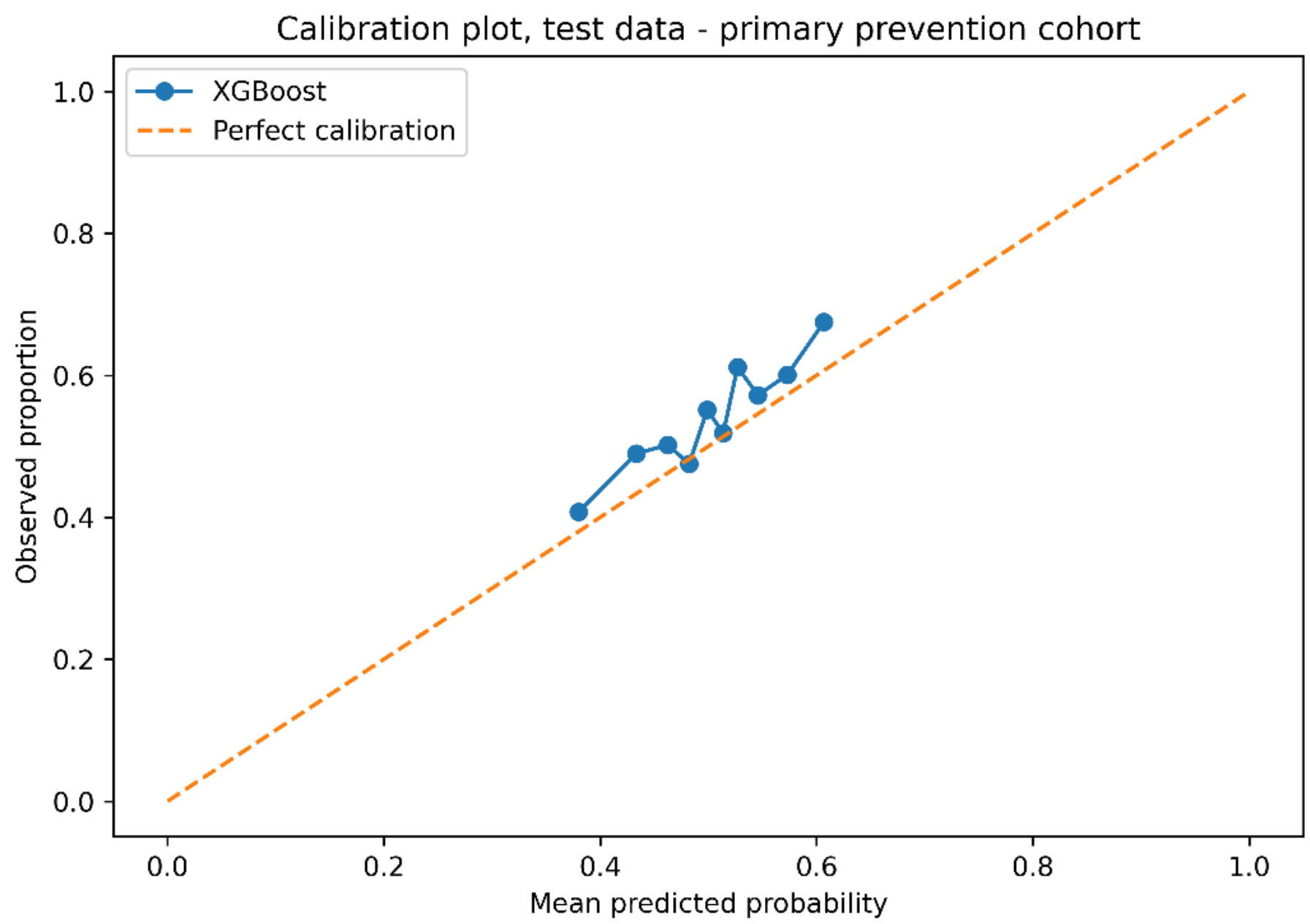


**Supplementary Figure S1**. Calibration plots for the full cohort (A) and the primary prevention cohort (B). The plots show imperfect calibration, with a tendency toward underprediction of adherence, particularly in the full cohort. This supports interpreting the XGBoost models as exploratory tools for variable ranking rather than as calibrated individual prediction models.

**Confounder selection**

| Exposure | Sex | Age | Marital status | Educational status | Income | Country of birth | Smoking | Body mass index | Waist circumference | Blood pressure | HbA1c | Triglycerides | LDL cholesterol | Total cholesterol | Comorbidities | Medications | Non-insulin antidiabetic | ACE inhibitor | Antithrombotic therapy | Dosetype | Statin intensity | Type of statin | Year of statin initiation | Statin initiation setting * | Primary health care centre ** |
|---|---|---|---|---|---|---|---|---|---|---|---|---|---|---|---|---|---|---|---|---|---|---|---|---|---|
| Antithrombotic therapy | Adjust | Adjust | Adjust | Adjust | Adjust | Adjust | Adjust | Adjust | Adjust | Do not adjust | Not included due to missing data | Do not adjust | Do not adjust | Do not adjust | Do not adjust | Do not adjust | Do not adjust | Do not adjust | Not included | Do not adjust | Do not adjust | Do not adjust | Adjust | Do not adjust | Adjust |
| Primary health care centre | Adjust | Adjust | Adjust | Adjust | Adjust | Adjust | Do not adjust | Do not adjust | Do not adjust | Do not adjust | Not included due to missing data | Do not adjust | Do not adjust | Do not adjust | Do not adjust | Do not adjust | Do not adjust | Do not adjust | Do not adjust | Do not adjust | Do not adjust | Do not adjust | Adjust | Do not adjust | Not included |
| Age | Adjust | Not included | Adjust | Adjust | Adjust | Adjust | Do not adjust | Do not adjust | Do not adjust | Do not adjust | Not included due to missing data | Do not adjust | Do not adjust | Do not adjust | Do not adjust | Do not adjust | Do not adjust | Do not adjust | Do not adjust | Do not adjust | Do not adjust | Do not adjust | Adjust | Do not adjust | Adjust |
| Type of statin | Adjust | Adjust | Adjust | Adjust | Adjust | Adjust | Adjust | Adjust | Adjust | Adjust | Not included due to missing data | Adjust | Adjust | Adjust | Adjust | Adjust | Adjust | Adjust | Adjust | Adjust | Do not adjust | Not included | Adjust | Do not adjust | Adjust |
| Country of birth | Adjust | Adjust | Do not adjust | Do not adjust | Do not adjust | Not included | Do not adjust | Do not adjust | Do not adjust | Do not adjust | Not included due to missing data | Do not adjust | Do not adjust | Do not adjust | Do not adjust | Do not adjust | Do not adjust | Do not adjust | Do not adjust | Do not adjust | Do not adjust | Do not adjust | Adjust | Do not adjust | Adjust |
| Non-insulin antidiabetic | Adjust | Adjust | Adjust | Adjust | Adjust | Adjust | Adjust | Adjust | Adjust | Do not adjust | Not included due to missing data | Do not adjust | Do not adjust | Do not adjust | Do not adjust | Do not adjust | Not included | Do not adjust | Do not adjust | Do not adjust | Do not adjust | Do not adjust | Adjust | Do not adjust | Adjust |
| Anxiety diagnosis | Adjust | Adjust | Do not adjust | Do not adjust | Do not adjust | Adjust | Do not adjust | Do not adjust | Do not adjust | Do not adjust | Not included due to missing data | Do not adjust | Do not adjust | Do not adjust | Do not adjust | Do not adjust | Do not adjust | Do not adjust | Do not adjust | Do not adjust | Do not adjust | Do not adjust | Adjust | Do not adjust | Adjust |
| ACE inhibitor | Adjust | Adjust | Adjust | Adjust | Adjust | Adjust | Adjust | Adjust | Adjust | Do not adjust | Not included due to missing data | Do not adjust | Do not adjust | Do not adjust | Do not adjust | Do not adjust | Do not adjust | Not included | Do not adjust | Do not adjust | Do not adjust | Do not adjust | Adjust | Do not adjust | Adjust |
| Marital status | Adjust | Adjust | Not included | Adjust | Do not adjust | Adjust | Do not adjust | Do not adjust | Do not adjust | Do not adjust | Not included due to missing data | Do not adjust | Do not adjust | Do not adjust | Do not adjust | Do not adjust | Do not adjust | Do not adjust | Do not adjust | Do not adjust | Do not adjust | Do not adjust | Adjust | Do not adjust | Adjust |
| Statin intensity | Adjust | Adjust | Adjust | Adjust | Adjust | Adjust | Adjust | Adjust | Adjust | Adjust | Not included due to missing data | Adjust | Adjust | Adjust | Adjust | Adjust | Adjust | Adjust | Adjust | Adjust | Not included | Do not adjust | Adjust | Do not adjust | Adjust |

Confounder: adjust | Mediator: do not adjust | Not included

**Supplementary Figure S2.** Confounder selection for the multilevel regression models in the full study cohort (n = 30 497) according to the modified disjunctive cause criterion. ACE: angiotensin-converting enzyme. LDL: low-density lipoprotein. Dosetype: statin dispensed through a multi-dose drug dispensing system (Pascal). *Statin initiation in primary vs. secondary care. **Included as a cluster variable.

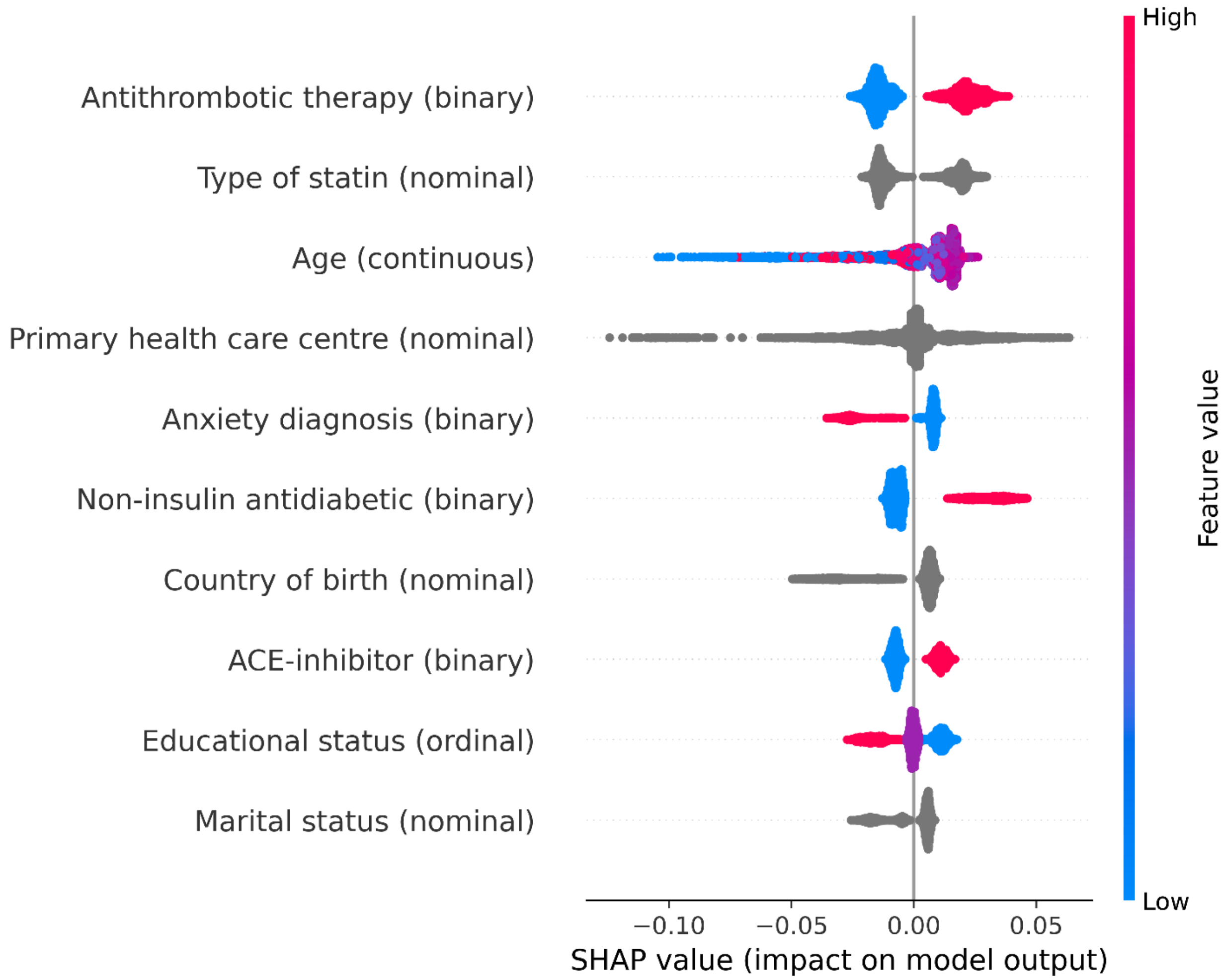


**Supplementary Figure S3.** SHAP values for the full study cohort (test data) illustrating the ten baseline characteristics most strongly associated with predicted adherence, defined as proportion of days covered (PDC) treated as a continuous variable. ACE: angiotensin-converting enzyme.

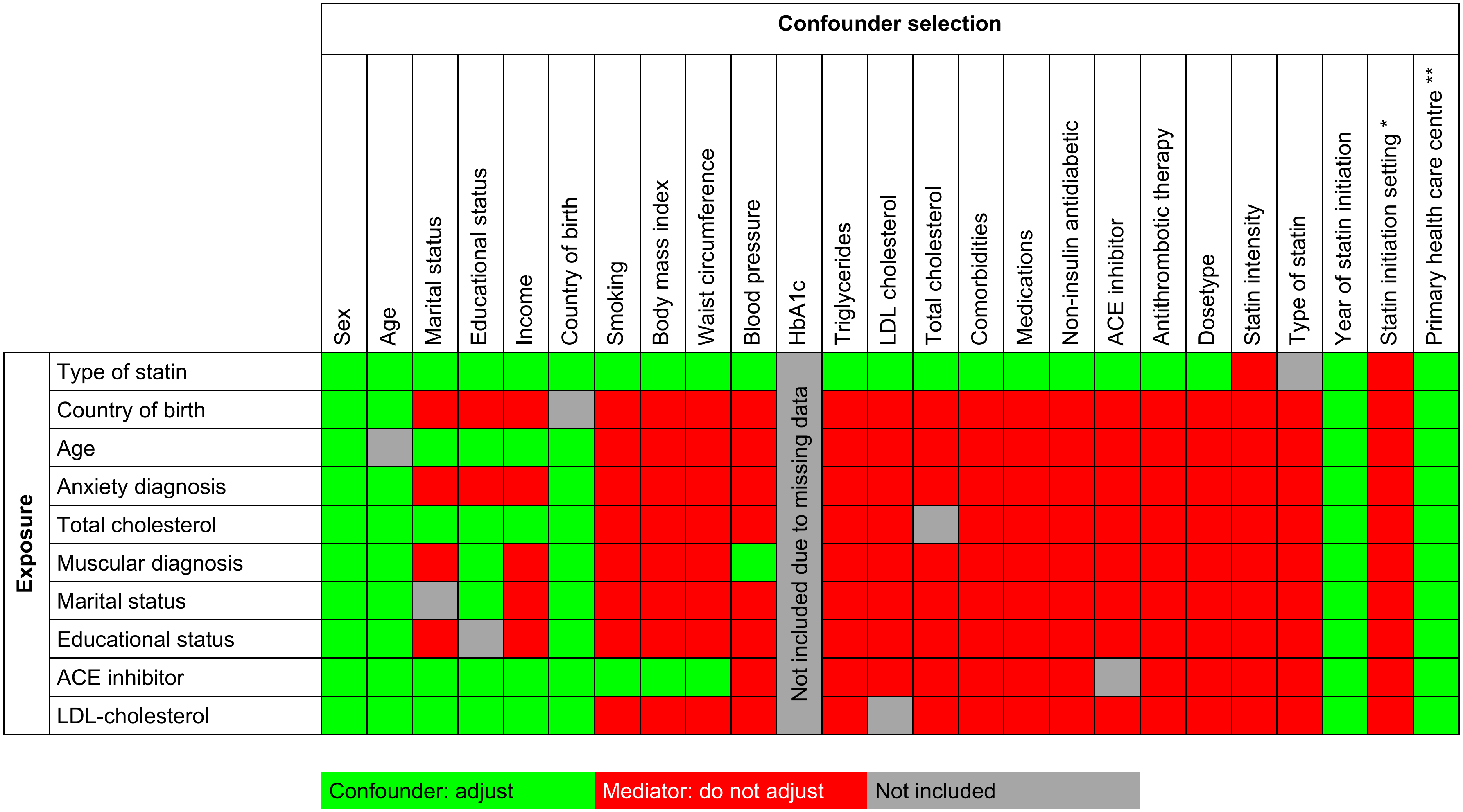


**Supplementary Figure S4.** Confounder selection for the multilevel regression models in the primary prevention group (n = 12 136) according to the modified disjunctive cause criterion. ACE: angiotensin-converting enzyme. LDL: low-density lipoprotein. Dosetype: statin dispensed through a multi-dose drug dispensing system (Pascal). *Statin initiation in primary vs. secondary care. **Included as a cluster variable.

## References


1. Ludvigsson JF, Andersson E, Ekbom A, Feychting M, Kim JL, Reuterwall C, et al. External review and validation of the Swedish national inpatient register. *BMC Public Health* 2011;**11**:450.
2. Ludvigsson JF, Svedberg P, Olén O, Bruze G, Neovius M. The longitudinal integrated database for health insurance and labour market studies (LISA) and its use in medical research. *Eur J Epidemiol* 2019;**34**:423-437.
3. Brooke HL, Talback M, Hornblad J, Johansson LA, Ludvigsson JF, Druid H, et al. The Swedish cause of death register. *Eur J Epidemiol* 2017;**32**:765-773.
4. Chen T, Guestrin C. XGBoost: A scalable tree boosting system. Proceedings of the 22nd ACM SIGKDD International Conference on Knowledge Discovery and Data Mining. 2016:785–794.
